# Enhanced *Nicotiana benthamiana* immune responses caused by heterologous plant genes from *Pinellia ternata*

**DOI:** 10.1186/s12870-018-1598-5

**Published:** 2018-12-17

**Authors:** Hafiz Muhammad Khalid Abbas, Jingshu Xiang, Zahoor Ahmad, Lilin Wang, Wubei Dong

**Affiliations:** Department of Plant Pathology, College of Plant Science and Technology and the Key Lab of Crop Disease Monitoring & Safety Control in Hubei Province, Huazhong Agricultural University, Wuhan, 430070 Hubei Province China

**Keywords:** Durable disease resistance, Hypersensitive response, *Nicotiana benthamiana*; non-self-recognition, Pathogenesis related genes, *Pinellia ternata*

## Abstract

**Background:**

*Pinellia ternata* is a Chinese traditional medicinal herb, used to cure diseases including insomnia, eclampsia and cervical carcinoma, for hundreds of years. Non-self-recognition in multicellular organisms can initiate the innate immunity to avoid the invasion of pathogens. A design for pathogen independent, heterosis based, fresh resistance can be generated in F_1_ hybrid was proposed.

**Results:**

By library functional screening, we found that *P. ternata* genes, named as *ptHR375* and *ptHR941*, were identified with the potential to trigger a hypersensitive response in *Nicotiana benthamiana*. Significant induction of ROS and Callose deposition in *N. benthamiana* leaves along with activation of pathogenesis-related genes viz.; *PR-1a*, *PR-5*, *PDF1.2*, *NPR1*, *PAL*, *RBOHB* and *ERF1* and antioxidant enzymes was observed. After transformation into *N. benthamiana*, expression of pathogenesis related genes was significantly up-regulated to generate high level of resistance against *Phytophthora capsici* without affecting the normal seed germination and morphological characters of the transformed *N. benthamiana*. UPLC-QTOF-MS analysis of *ptHR375* transformed *N. benthamiana* revealed the induction of Oxytetracycline, Cuelure, Allantoin, Diethylstilbestrol and 1,2-Benzisothiazol-3(2H)-one as bioactive compounds. Here we also proved that F_1_ hybrids, produced by crossing of the *ptHR375* and *ptHR941* transformed and non-transformed *N. benthamiana*, show significant high levels of *PR*-gene expressions and pathogen resistance.

**Conclusions:**

Heterologous plant genes can activate disease resistance in another plant species and furthermore, by generating F_1_ hybrids, fresh pathogen independent plant immunity can be obtained. It is also concluded that *ptHR375* and *ptHR941* play their role in SA and JA/ET defense pathways to activate the resistance against invading pathogens.

**Electronic supplementary material:**

The online version of this article (10.1186/s12870-018-1598-5) contains supplementary material, which is available to authorized users.

## Background

Non-self-recognition is one of the most important phenomena to initiate the innate immunity in multicellular organisms. It helps to prevent the invasion of pathogens and to maintain the genetic polymorphism of organisms [1]. All kinds of organisms have the ability to recognize foreign DNA, RNA or proteins of invading pathogens. Non-self-recognition is the event of early stages of parasites’ invasion and plays an important role to overcome the infection by pathogens, and it occurs among all organisms as basic characteristics of the cell to cell interaction [2, 3]. In plants, induced disease resistances are triggered by pathogen infection, which is dependent on the non-self-recognition. As a result of non-self-recognition, phytoalexins and other cell wall strengthening materials are produced around the infection site to inhibit the penetration of pathogen [4].

It is considered that the mutual interactions between host and pathogen are the major drivers for their co-evolution. A pathogen can be more virulent in the presence of a variety of hosts instead of single kind of host and vice versa. The way of pathogen attack is the key factor to determine the mechanism of host defense [5]. The activation of defense response in plants includes sudden death of plant cells known as hypersensitive response (HR) [6], a burst of reactive oxygen species (ROS) [7], activation of defense-related genes [8] and production of antimicrobial compounds e.g. phytoalexins [9]. In the non-inoculated portion of plants, plants have the ability to develop systemic resistance in addition to local resistance. This kind of resistance usually divided into two groups: systemic acquired resistance (SAR) and induced systemic resistance (ISR) [10]. Accumulation of salicylic acid (SA) and induction of pathogenesis-related (PR) genes are involved in the establishment of SAR. Moreover, SAR is long lasting and have potential to inhibit the broad spectrum of pathogens such as fungi, bacteria, nematodes and viruses [11]. While on the other hand, ISR is dependent on the plant roots colonization by plant growth promoting bacteria and Jasmonic acid (JA) and ethylene (ET) signaling [12].

For the development of resistant crops, understanding with basic mechanism of host-pathogen interaction is necessary. It is divided into two types, incompatible and compatible interactions leading to accelerating the resistance and susceptibility to certain diseases, respectively. Plant breeders use incompatible interaction as a basic tool for the development of resistant cultivars in the sustainable agricultural system. The activation of incompatible interaction is mainly dependent on the presence of two-layered plant immune systems to protect them against a variety of pathogens. The first layer, pathogen-associated molecular patterns (PAMP) trigger immunity, is activated upon the recognition of the pathogen by PRR (pattern recognition receptors), while the second layer, effector trigger immunity (ETI), is activated by the pathogen effectors (Avr) recognized by R-genes of the host plant [13]. As this R-gene recognizes the specific Avr gene and then initiate the immune response, so it is also known as R-gene mediated immunity [14, 15]. As the R-gene of the host is a basic selection force on corresponding pathogen *Avr* gene which results in the modification of *Avr* gene to overcome the effect of old R gene of the host. This host-pathogen evolution can explain that how one cultivar loses its resistance in field [16, 17]. By keeping in view, it is the basic need of time to develop some new and unique strategies to introduce long-term and broad-spectrum resistance against potential pathogens.

Heterosis, also known as hybrid vigor, is the phenomenon which describes the vigor of F_1_ heterozygous hybrid more than that of their homozygous parents. It can explain the increased plant biomass, yield, fertility, development potential and disease resistance [18]. For the better understanding of heterosis, three different genetic hypothesis, dominance, overdominance and epistasis, have been proposed [18, 19]. Heterosis has been widely used to enhance the yield and productivity of plants [20–22].

In our study, we proposed that pathogen triggered plant immunity is a specific form and result of non-self-recognition. We also proposed that pathogen infection is a driving force for the formation and evolvement of non-self-recognition. In the evolution of species formation, without pathogen, it might not have non-self-recognition, furthermore, some species boundary might be blur. Non-self-recognition might be used to develop pathogen independent plant resistance. Based on these hypotheses, we designed this research and identified some genes from *P. ternata*, which can initiate the local and systemic defense responses in *N. benthamiana* through the mechanism of non-self-recognition. For further characterization, these genes were cloned and transferred into *N. benthamiana*, and found that they can increase tobacco basal resistances.

## Methods

### Plant materials and pathogen cultures

*P. ternata* was collected from Wuhan Botanical Garden, Wuhan, Hubei Province, P. R. China and grown in a controlled growth chamber at 20–26 °C and 14 h/10 h of light/dark conditions. *N. benthamiana* and *Lycopersicon esculentum* seeds were germinated on nutrient soil and transplanted individually in pots under controlled growth conditions of 24–28 °C and 16 h/8 h light and dark intervals. *Gossypium hirsutum* was grown on nutrient soil under controlled conditions at 24–28 °C and 16 h/8 h light and dark intervals. *Pectobacterium carotovora* was maintained on Luria-Bertani (LB) medium (10 g tryptone, 5 g yeast extract, 10 g NaCl per liter distilled water) at 28 °C in the dark. *Phytophthora capsici* (LT263)*,* procured from the Key Lab of Crop Disease Monitoring and Safety Control in Hubei Province, Huazhong Agricultural University, Wuhan 430,070, China, was maintained on V8 (100 ml V8 juice, 1 g CaCO_3_ per liter distilled water) media at 25 °C under dark conditions.

### Construction of *P. ternata* cDNA library

*P. ternata* was inoculated with *P. carotovora* for the construction of cDNA library. The tuber of *P. ternata* was cleaned and soaked into *P. carotovora* liquid, which was incubated at 28 °C for 24 h before use, for 10 min. Leaf samples were collected at different time intervals (24, 36 and 48 h), frozen in liquid nitrogen and stored at − 80 °C.

Total RNA from collected samples was extracted with the Trizol reagent. After that, the mRNA was purified from total RNA using PolyATtract® mRNA isolation systems (Promega). The cDNA library was created using PrimeScript™ double strand cDNA synthesis kit (TaKaRa) with specific Oligo dT primer (containing *Xba* I cleavage site). Three pairs of adaptor containing *Sac* I cleavage sites were added to cDNA library. Then the library was digested with *Xba* I and *Sac* I enzyme at the same time. The short fragments were removed by AxyPrep PCR cleanup kit (AxyGEN). Afterwards, the product was ligated into pTRV2Ex vector [23] (*Xba* I and *Sac* I enzymes were used to digest vector in advance). The ligation product was subsequently transformed into Trans DH5α *E. coli* cells. Plasmids of *E. coli* cells were extracted using EasyPure® Plasmid MiniPrep Kit (TransGen) and then transformed into *A. tumefaciens* strain EHA105. Individual colonies were picked with toothpicks and incubated at 28 °C for overnight. Colony PCR was performed using primers GF (5’-TACAGGTTACTGAATCACTTGCGCTA-3′) and GR (5’-CCGTAGTTTAATGTCTTCGGGACA-3′) to confirm the cDNA library quality and then saved at − 80 °C.

### Functional screening and sequence analysis of cDNA library

For functional screening of cDNA library of *P. ternata*, Agrobacterium containing pTRV_1_, pTRV_2_Ex empty vector, pTRV_2_Ex::target gene, respectively, were grown overnight at 28 °C in LB containing kanamycin (50 mg/L) and rifampicin (50 mg/L). Agrobacterium cells were collected by centrifugation at 4000 rpm for 10 min, re-suspended in MMA solution (10 mmol/L MgCl_2_, 10 mmol/L MES, 20 g/L sucrose, 100 mmol/L acetosyringone, PH = 5.6), adjusted to final OD_600_ = 0.8–1.0 and left at room temperature for 3 h without shaking. The pTRV1 solution and target gene solution were mixed 1:1, and pressure-infiltrated into the leaf of *N. benthamiana* plant using a 1 mL syringe without a needle. *Avr*4-*Cf*4 was used as positive control while MMA buffer and empty vector were used as negative control. Symptom variation of injected leaves was observed and recorded every day.

Colonies repeatedly showing HR symptoms on plants were sequenced by Wuhan AuGCT (http://wh.augct.com/) using GR primer. NCBI (National Center for Biotechnology Information) BLAST search was performed to determine the homologs of the colonies.

### Staining for ROS accumulation and callose deposition

The 4–5-weeks old *N. benthamiana* plants were used to study ROS accumulation and callose deposition. Leaves of tobacco plants were infiltrated with *ptHR* genes and empty vector as control. At the start of symptoms development, leaves were collected to detect ROS and callose. ROS staining was performed by using DAB kit (CWBIO). Briefly, leaves samples were incubated with DAB solution mixture (1 mL reagent A and 50 μl reagent B) in dark for 1 h and then boiled in 95% ethanol for 30 min to remove chlorophyll. The ROS accumulates were observed under Nikon eclipse 55i optical microscope. This experiment was repeated thrice with three replicates.

Callose staining was performed according to the method described by [24]. In brief, tobacco leaves were treated with Buffer I (90 mmol/L Na_2_HPO_4_, 5 mmol/L citric acid and 1% glutaraldehyde, pH 7.4) for overnight. After that leaves were washed with ddH_2_O and 95% ethyl alcohol was used to soak leaves, then put into boiling water to clear chlorophyll. Sequentially transparent leaves were washed with Buffer II (50% ethyl alcohol, 67 mmol/L Na_2_HPO_4_, pH 12.0) and then staining was performed for 1 h in dark at room temperature with staining buffer (0.1% aniline blue, 67 mmol/L Na_2_HPO_4_, pH 12.0). Callose deposits were observed under Nikon eclipse 80i ultraviolet epifluorescence.

### Analysis of antioxidants activity

Leaves of 4–5 weeks old tobacco plants were infiltrated with *ptHR* genes and empty vector as control. Samples were collected at different time intervals (0, 24, 48, 72, 96, 120, 144 and 168 h) after infiltration, frozen in liquid nitrogen and stored at − 80 °C. Polyphenol oxidase (PPO), Peroxidase (POD) and Superoxide dismutase (SOD) were measured according to the methods of [25–27], respectively.

### Analysis of the relative expression of pathogenesis-related genes induced by *ptHR* genes

To analyze the relative expression of pathogenesis-related genes induced by *ptHR* genes in *N. benthamiana*, leaves of *N. benthamiana* were infiltrated with *ptHR* genes and empty vector as control. Samples were collected at different time intervals (0, 24, 48, 72, 96 and 120 h), frozen in liquid nitrogen and saved at − 80 °C for further use. Total RNA was extracted with the Trizole reagent. cDNA was synthesized by using HiFiScript Quick gDNA Removal cDNA kit (CWBIO) and concentrations were adjusted to be equal. Real-time quantitative PCR (RT-qPCR) was performed to study the relative expression of several pathogenesis-related genes, by using SYBR® Premix Ex TaqTMII (TliRNaseH Plus) (TaKaRa Clontech). The specific primers (Additional file 1: Table S1) were used from [7, 8]. *EF-1α* was used as reference gene. PCR mixture was processed by using CFX96™ Real-Time PCR Detection System (BIO-RAD), under the following program: 95 °C for 30 s followed by 40 cycles of 95 °C for 5 s and 60 °C for 30 s. A melting curve was established from 65 °C to 95 °C. Three replicates were used for amplification from each treatment and also for control. The 2^-∆∆CT^ method [28] was used to quantify the relative expression of defense-related genes.

### Construction of pCAMBIA3301 expression vector

*ptHR375* and *ptHR941* were cloned into pCAMBIA3301 binary expression vector [29] between *Bgl* II-*BstE* II enzyme sites. TF primer, which contains *Bgl* II enzyme site (5’-ACTGGAAGATCTTACAGGTTACTGAATCACTTGCGCTA-3′) and TR primer (5′- ATAGATGGTNACCCCGTAGTTTAATGTCTTCGGGAC-3′), which contains *BstE* II enzyme site were used to amplify *ptHR* genes in pTRV2 Ex vector. Double digestion was subsequently performed on amplified genes. The product was ligated into corresponding sites of the pCAMBIA3301 vector in place of the *GUS*-containing region. The correct insertions of *ptHR* genes were confirmed by DNA sequence analysis. Finally, the constructs were introduced into *A. tumefaciens* EHA105 strain.

### Generation of transgenic plants expressing *ptHR* genes

Transgenic *N. benthamiana* plants expressing *ptHR375* and *ptHR941* genes were obtained by using *Agrobacterium*-mediated leaf disc transformation method [30]. Transgenic T_1_ *N. benthamiana* seeds were selected on MS media containing bialaphos (3 mg/L), verified by PCR and sequence analysis.

### Controlled pollination to develop F_1_ hybrid

Female flowers were emasculated at stage 11 [31] of flower development to avoid self-pollination, and hand pollination was performed with pollens collected from male flowers at anthesis. After artificial pollination, flowers were labeled accordingly and covered with 5 cm soda drinking straw enclosed at one end. About 60 h after pollination, corolla fell down together with protective soda drinking straw. After 20–25 days of pollination, seeds were collected and sun-dried [32].

### Engineered defense responses protect tobacco plant against fungal pathogen

Leaves were detached from 3 to 4 weeks old T_3_ generation of transformed *N. benthamiana* and F_1_ hybrid plants, and placed in a petri dish lined with a tissue moistened with sterile water. Detached leaves from non-transformed *N. benthamiana* were used as a control. Leaves were inoculated with *P. capsici* by using the method of [33]. The diameter of lesions was measured at 48 h after inoculation and inhibition ratio was calculated with the formula: [(control lesion diameter – treatment lesion diameter) / (control lesion diameter – pathogen disk diameter)] × 100.

### Penalty test of *ptHR* genes on *N. benthamiana*

To observe the effect of *ptHR375* and *ptHR941* genes on seed germination and morphological characters, 30 seeds of each, non-transformed and transformed *N. benthamiana* were grown on petri plates lined with moistened filter paper. After 10 days, percent germination was calculated by using the formula: (No of seeds germinated/total No of seeds sown) × 100. Morphological characters were measured after 15 days and averages were calculated.

### UPLC-QTOF-MS analysis of transformed *N. benthamiana*

For the analysis of different metabolites, the samples were prepared according to the method described by [34]. For the LC-MS analysis, Waters Ultra Performance Liquid Chromatography system was used according to the method previously described by [35]. Non-transformed *N. benthamiana* was used as a control. Ribitol (50 μg/mL), Sigma Aldrich, USA (Cat#A9790) was used as internal standard. The raw data were processed by using the MZmine2 software.

## Results

### Screening of *P. ternata* genes inducing hypersensitive responses in *N. benthamiana*

**Fig. 1:**
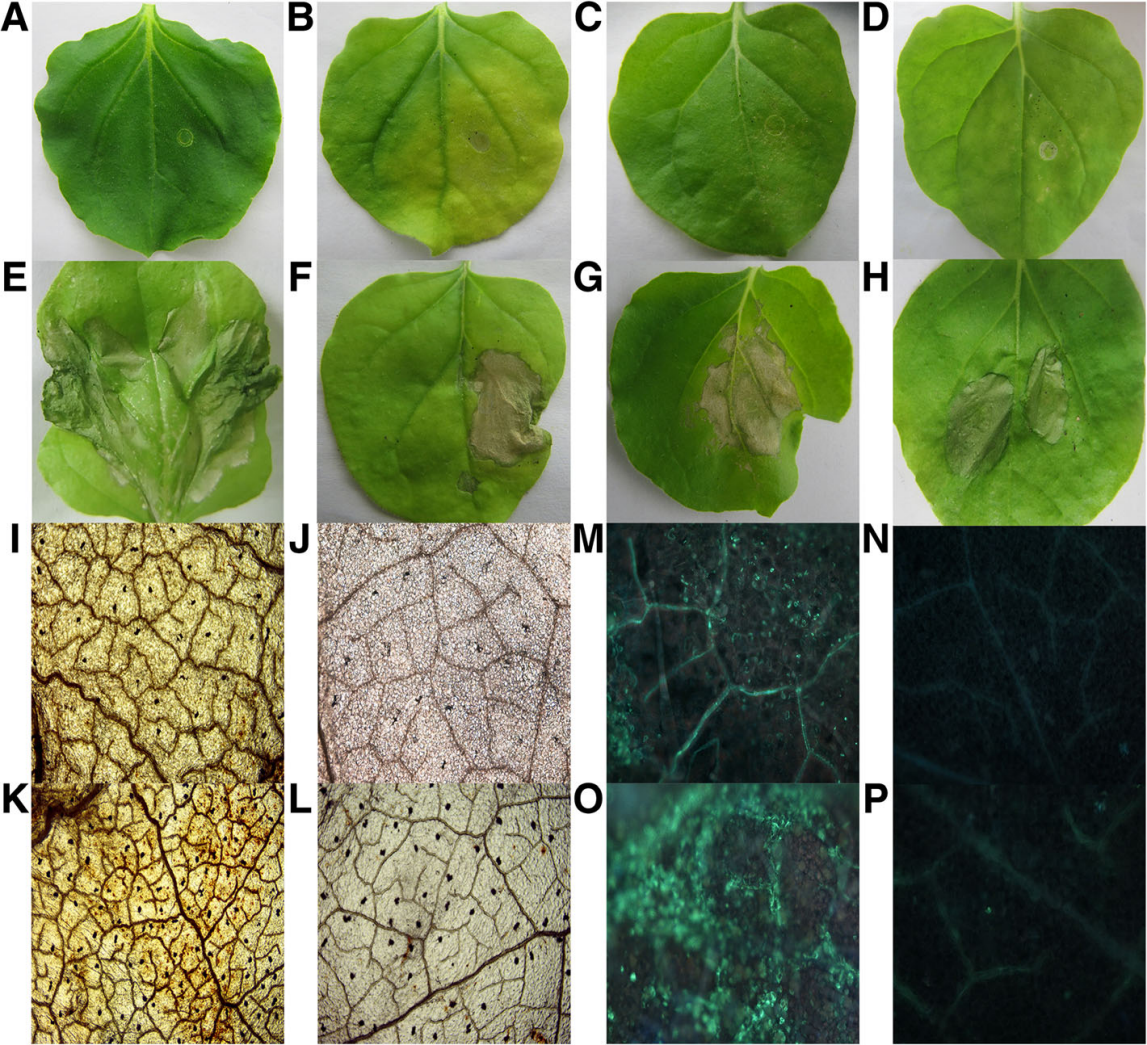
Hypersensitive response, ROS burst and callose deposition induced by *ptHR* genes in *N. benthamiana* leaves. *N. benthamiana* leaves were infiltrated with buffer, EHA105 Agrobacterium strain, empty vector, pTRV_2_-*Avr*4 and pTRV_2_-*Cf*4 as positive control, and *ptHR* genes. Pictures were taken at 48 hpi. **a-h** HR induced by *ptHR* genes in *N. benthamiana* leaves treated with **a** buffer, **b** Agrobacterium EHA105 strain, **c** pTRV empty vector, **d** pTRV_1_ as a control, **e** pTRV_2_-*Avr*4 and pTRV_2_-*Cf*4 as a positive control, **f** *ptHR941*, **g** *ptHR375,* and **h** *ptHR293*. **i-l** ROS accumulation induced by *ptHR* genes in *N. benthamiana* leaves treated with, **i** *ptHR941* cloned in pTRV Ex vector, **j** pTRV empty vector, **k** *ptHR941* cloned in pCAMBIA3301 vector, **l** pCAMBIA3301 empty vector. **m-p** Callose deposition induced by *ptHR* genes in *N. benthamiana* leaves treated with, **m** *ptHR941* cloned in pTRV Ex vector, **n** pTRV empty vector, **o** *ptHR941* cloned in pCAMBIA3301 vector, **p** pCAMBIA3301 empty vector. *N benthamiana* leaves were infiltrated with *ptHR* genes and empty vector (pTRV and pCAMBIA3301). Each experiment was repeated three times, and each time the same results were observed

Complementary DNA reverse transcripts from total RNA of *P. ternata* were ligated into a binary pTRV expression vector and transformed into *A. tumefaciens* EHA105 strain. A total of 1268 cDNA colonies were selected, cultured and saved at − 80 °C. To check the quality of this library, 100 random cDNA colonies were selected to perform colony PCR, and it was observed that 95% of the recombinant colonies inserts ranged from 100 to 1000 bp and seldom repeated (Additional file 1: Figure S1). Each individual *A. tumefeciens* colony, 1268 colonies in total, was infiltrated into *N. benthamiana, Lycopersicon esculentum* and *Gossypium hirsutum* leaves with a 1 mL needleless syringe. Typical HR symptoms were observed around infiltration sites at the 48 h of post infiltration as compared to empty vector and buffer which were used as negative control (Fig. 1a-h, Additional file 1: Figure S2a-h). In total, 49 cDNA colonies can repeatedly induce typical HR symptoms, which indicates that 3.86% (49/1268) of *P. ternata* genes can cause non-self recognition in *N. benthamiana*.

After the transformation of *ptHR* genes into a pCAMBIA3301 vector, Agrobacterium-mediated transient expression was performed to confirm the typical HR symptoms on *N. benthamiana* and *L. esculentum* leaves. It was clearly observed that these genes induced HR symptoms on *N. benthamiana* and *L. esculentum* (Additional file 1: Figure S2i-o). *P. ternate* leaves were also injected **(**Additional file 1: Figure S2p-q) with these genes to verify the non-self-recognition, and it was found that these genes couldn’t activate the defense mechanism of parent source. During our initial screening, *ptHR375* and *ptHR941* showed very promising and consistent results related to our objective, so we selected these 2 genes for further experiments.

### Homology analysis of *P. ternata* genes

Forty-nine different colonies, repeatedly inducing HR symptoms on *N. benthamiana* leaves, were selected for sequencing. NCBI analysis of sequences revealed that 25 sequences showed homology to known proteins with identity from 70 to 96% **(**Additional file 1: Table S2), 5 sequences shared homology with transcription factors **(**Additional file 1: Table S3), and remaining 19 shared no homology to known proteins considered to be novel **(**Additional file 1: Table S4). These newly identified genes were named as *ptHR* (*Pinella ternata* hypersensitive response) genes.

### *ptHR* genes induced ROS accumulation and callose deposition in *N. benthamiana* leaves

ROS burst and callose deposition, both are the early events of plant defense mechanism against invading pathogens and resist their penetration. ROS accumulation and callose deposition, both experiments were performed with pTRV and pCAMBIA3301 constructs. pTRV and pCAMBIA3301 empty vectors were used as controls for comparison. As compared to empty vector, it was observed clearly from the microscopic images that leaves treated with *ptHR* genes induced ROS accumulation (Fig. 1i-l) and callose deposition in *N. benthamiana* leaves (Fig. 1m-p).

### *ptHR* genes enhanced antioxidant activities in *N. benthamiana*

**Fig. 2:**
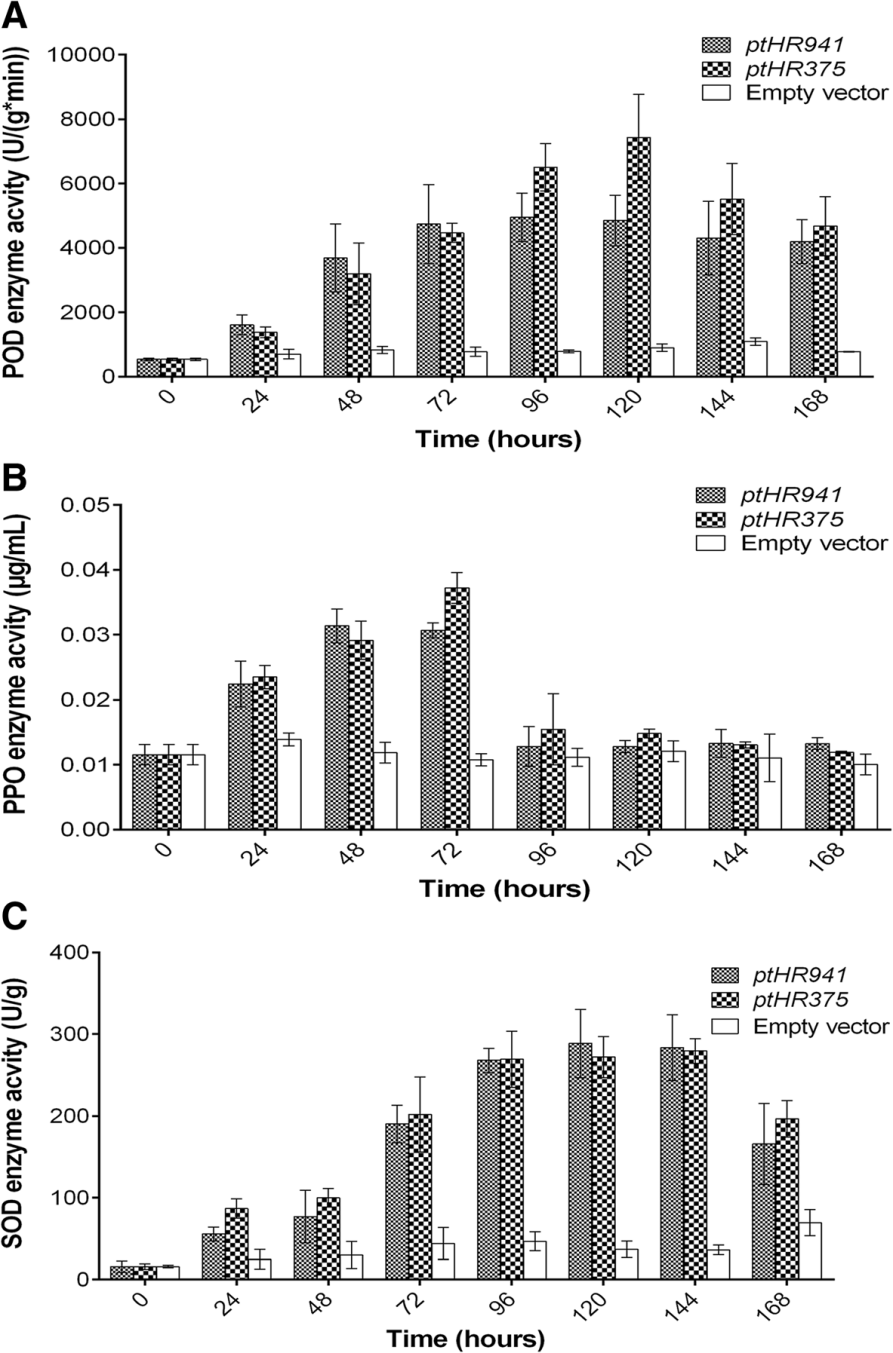
Analysis of antioxidant activities in *N. benthamiana* induced by *ptHR* genes. **a** POD activity under influence of *ptHR941* and *ptHR375*, **b** PPO activity under influence of *ptHR941* and *ptHR375*, and **c** SOD activity under influence of *ptHR941* and *ptHR375*. Results are mean values from three independent experiments. Vertical bars indicate SD

Antioxidants are the elements which can protect plants against a variety of pathogens, the plants which possess a level of resistance often have a certain amount of these elements. SOD, POD and PPO were measured from 0 to 168 h after infiltration of *ptHR* genes. In the case of POD, it was observed that its activity stimulated at 24 h after treatment, peaked at 96 h for *ptHR941*, and at 120 h for *ptHR375*, and then declined gradually (Fig. 2a). While in the case of PPO, maximum activity was observed at 48 h for *ptHR941* and at 72 h for *ptHR375*, and then started to decline down gradually (Fig. 2b). Similar case was observed with SOD where maximum activity was observed at 120 h for *ptHR941* and at 144 h for *ptHR375* (Fig. 2c), while control was lesser than the treatments.

### *ptHR* genes activated the expression of pathogenesis-related genes in *N. benthamiana*

**Fig. 3:**
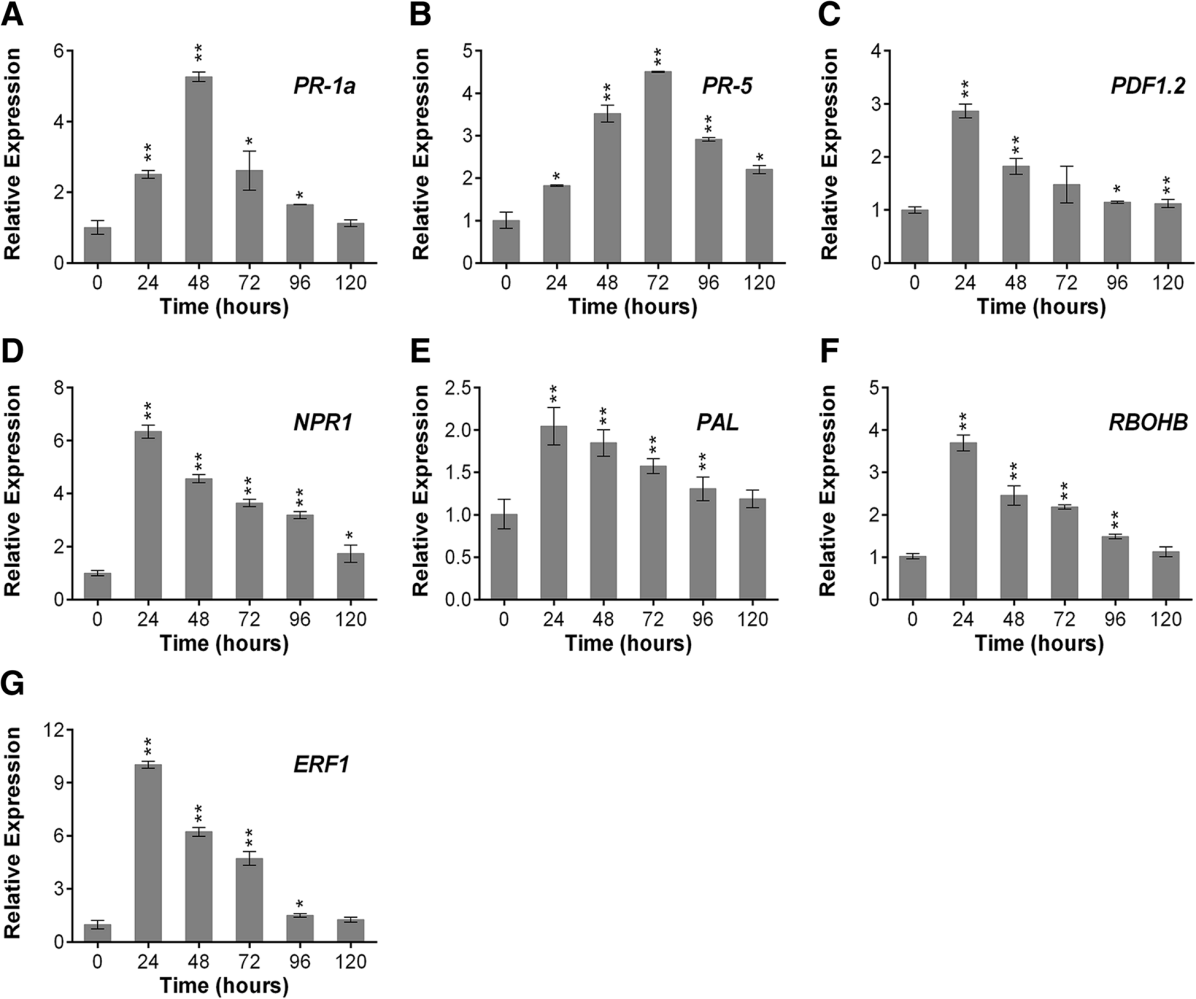
Relative expression levels of pathogenesis-related genes in *N. benthamiana. N. benthamiana* leaves were infiltrated with *ptHR941* and an empty vector as control for RT-qPCR analysis. Leaves infiltrated with empty vector were used as control for relative quantification of gene expression. *EF-1α* was used as internal control. **a-g** Relative expression levels of **a** *PR-1a* vs control, **b** *PR-5* vs control, **c** *PDF1.2* vs control, **d** *NPR1* vs control, **e** *PAL* vs control, **f** *RBOHB* vs control, and **g** *ERF1* vs control. Significance was determined by t-test: *P < 0.05, **P < 0.01. Results are the mean values from three independent experiments. Vertical bars indicate SD

To further study the mechanism of *ptHR* gene induced plant resistance, we analyzed the relative expression levels of pathogenesis-related genes, from SA and JA/ET pathways, of *N. benthamiana* infiltrated with *ptHR941* and *ptHR375* compared to empty vector **(**Fig. 3, Additional file 1: Figure S3). From the results, it was observed that *ptHR* genes were playing a significant role for the enhancement of plant defense by up-regulating the expression level of *PR*-genes. In the case of *N. benthamiana* plants infiltrated with *ptHR941*, it was observed that *PR-1a* and *PR-5* showed maximum up-regulation at 48 h and 72 h of post-infiltration, respectively, (Fig. 3a-b), and then started to decline. While at 24 h of post- infiltration, *PDF1.2, NPR1, PAL, RBOHB* and *ERF1* were showing maximum up-regulation **(**Fig. 3c, d, e, f and g). From the present results we speculated that *ptHR*941 and *ptHR*375 works in SA and JA/ET pathways to contribute in resistance mechanism.

### Generation of transgenic tobacco plants expressing *ptHR375* and *ptHR941*

**Fig. 4:**
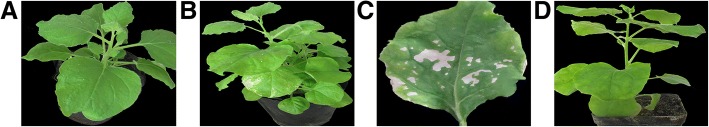
Typical phenotypic response of *ptHR* gene transformed *N. benthamiana.* **a** Non-transformed *N. benthamiana* plant, (**b**) and (**c**) transformed *N. benthamiana* plants with lesions caused by non-self-recognition of *ptHR* genes in T_0_ generation, and (**d**) T_1_ generation of transformed *N. benthamiana*. Lesions were disappeared in the T_1_ generation and with normal morphology

By using the Agrobacterium-mediated leaf disc transformation method, positive transgenic *N. benthamiana* plants were obtained (Fig. 4). A typical phenotypic variation was observed on leaves of transgenes as compared to non-transgene plants (Fig. 4b-c). From these variation, positivity of target gene integration was assumed, but for further confirmation, after the extraction of genomic DNA, PCR was performed with gene-specific primers and found that plants with variations all were positive and containing *ptHR* genes. Seeds were collected from positive plants and screened on MS media against Bialaphos antibiotic for the development of next generations.

### *N. benthamiana* expressing *ptHR* genes confers increased transcriptome levels of pathogenesis-related genes

**Fig. 5:**
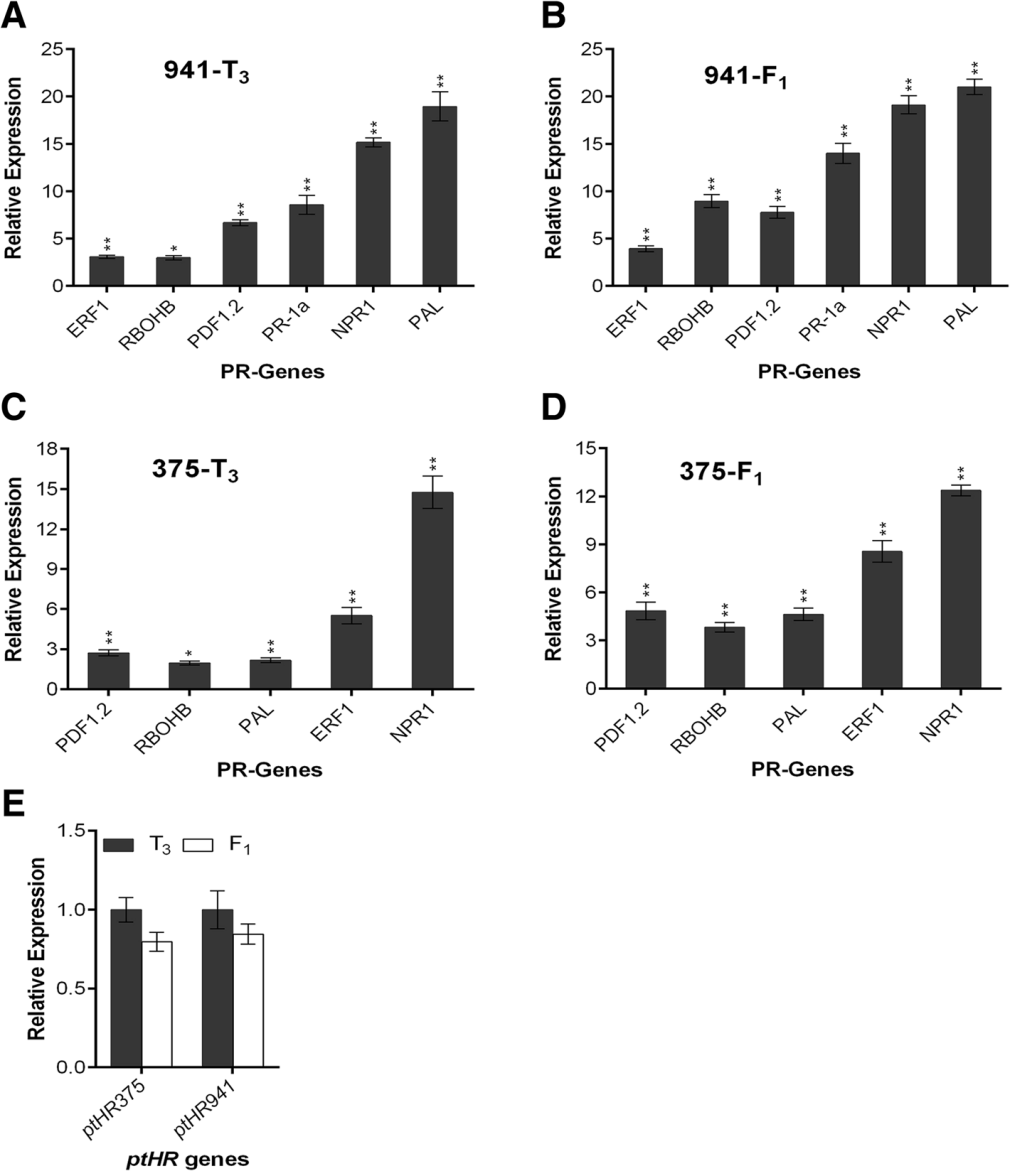
Relative expression levels of pathogenesis-related genes in transformed and F_1_ hybrid *N. benthamiana.* Leaves were sampled from transformed and F_1_ hybrid *N. benthamiana* to extract total RNA for RT-qPCR analysis. Non-transformed and transformed (T_3_) *N. benthamiana* were used as control for relative quantification of gene expression. *EF-1α* was used as internal control. **a** Relative expression of *PR*-genes in *ptHR941* transformed *N. benthamiana* compared with non-transformed *N. benthamiana* control, **b** Relative expression of *PR*-genes in *ptHR941*-F_1_ hybrid compared with transformed (T_3_) *N. benthamiana* control, **c** Relative expression of *PR*-genes in *ptHR375* transformed *N. benthamiana* compared with non-transformed *N. benthamiana* control, **d** Relative expression of *PR*-genes in *ptHR375*-F_1_ hybrid compared with transformed (T_3_) *N. benthamiana* control, and **e** Relative expression of *ptHR* genes in F_1_ hybrid compared with transformed (T_3_) *N. benthamiana* control. Results are the mean values from three independent experiments. Vertical bars indicate SD. Significance was determined by t-test: *P < 0.05, **P < 0.01

To study the resistance level, transcriptome levels of *PR*-genes were quantified in 12 independent transformed *N. benthamiana* lines relative to non-transformed *N. benthamiana*. For the confirmation of resistance level either it is inherited to the next generation or not, *PR*-gene expression level was measured up to T_3_ generation (Fig. 5). From the results it was observed that, in case of *ptHR941* and *ptHR375* transformed *N. benthamiana*, *PR*-genes were showing significant upregulation relative to their non-transformed *N. benthamiana* control (Fig. 5a and c). Significant expression of *ptHR941* and *ptHR375* genes was also observed in transformed *N. benthamiana* (Additional file 1: Figure S4). F_1_ hybrid was generated by performing the cross of T_3_ generations of transformed *N. benthamina* with non-transformed *N. benthamina,* and then *PR*-genes expression level was measured. It was observed from the results that all considered *PR*-genes in F_1_ hybrid showed significant upregulation relative to transformed (T3) *N. benthamiana* control (Fig. 5b and d), while the expression level of *ptHR* genes in F_1_ hybrid was non-significantly down regulated as compared to transformed (T_3_) *N. benthamiana* (Fig. 5e). From these results, it is considered that, in view of the phenomenon of heterosis, the F_1_ hybrid has obtained the considerable level of pathogen independent resistance by enhancing its *PR*-gene transcriptome levels.

### Engineered defense responses protect tobacco plant against fungal pathogen

**Fig. 6:**
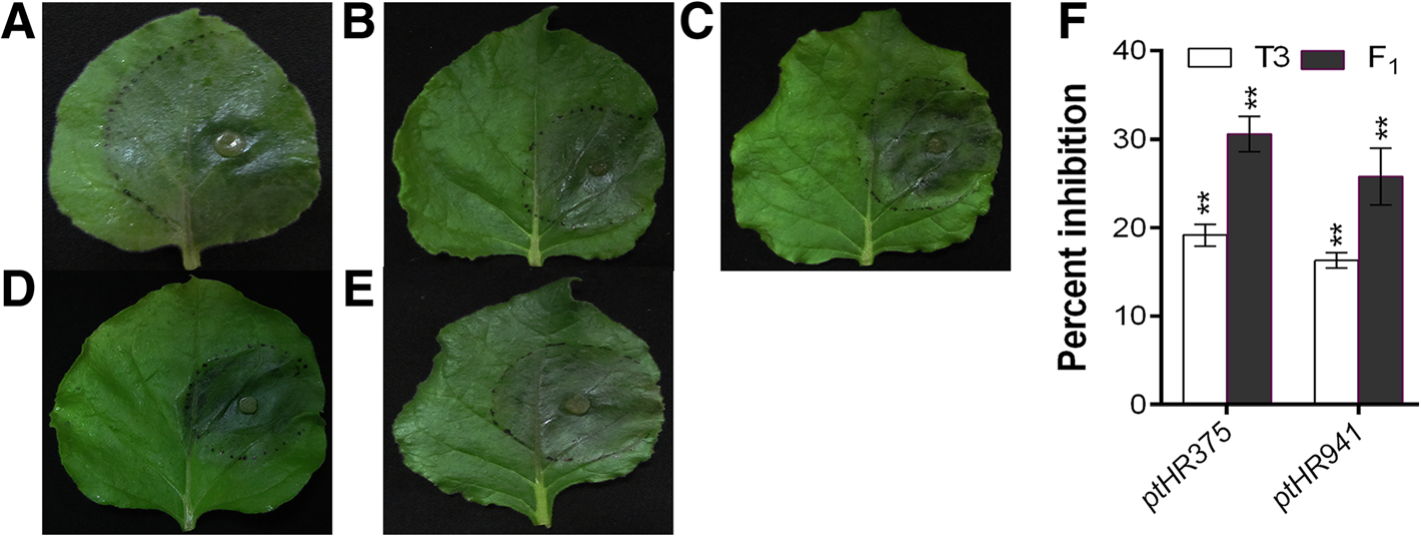
Resistant functions of *ptHR375* and *ptHR941* in transformed *N. benthamiana* and F_1_ hybrids. **a** Non-transformed *N. benthamiana* leaves (lesion diameter is 36 ± 0.086 mm) as control, **b** *ptHR375* transformed *N. benthamiana*, **c** *ptHR941* transformed *N. benthamiana,* **d** *ptHR375* F_1_ hybrid, **e** *ptHR941* F_1_ hybrid and **f** percent inhibition against *P. capsici* compared with control. Results are the mean values from three independent experiments. Vertical bars indicate SD. Significance was determined by t-test: **P < 0.01

Resistance acquired by transformed *N. benthamiana* in response to *ptHR375* and *ptHR941* was measured against fungal pathogen, *P. capsici*, in 12 independent lines. From the results, it was found that *ptHR375* and *ptHR941* played an important and significant role to inhibit the infection of *P. capsici* (Fig. 6). Results revealed that leaves of transformed *N. benthamiana* and F_1_ hybrids significantly restricted the growth of *P. capsici* as compared to non-transformed *N. benthamiana*.

### *N. benthamiana* expressing *ptHR375* and *ptHR941* showed normal morphology

**Fig. 7:**
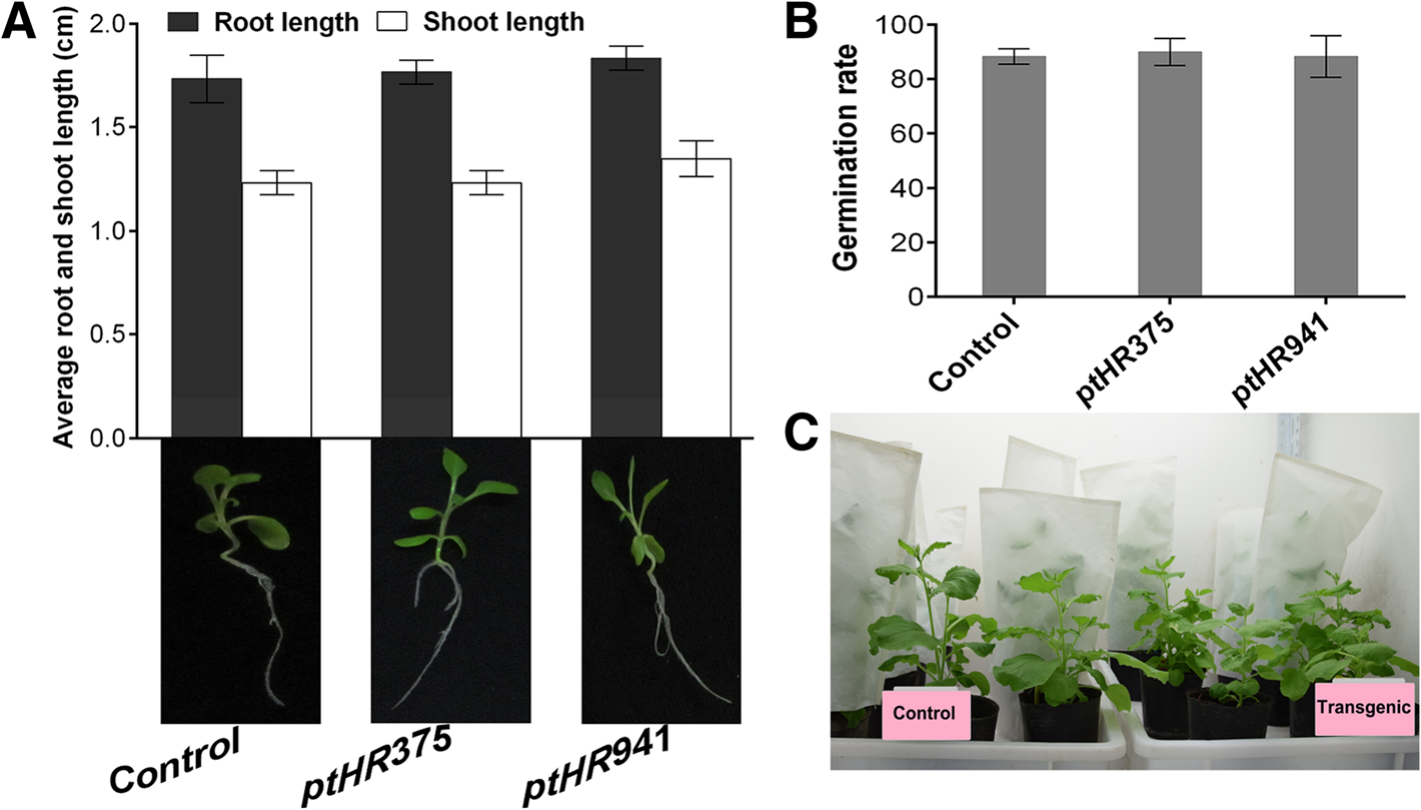
Germination and morphological characters of non-transformed and transformed *N. benthamiana.* **a** Average root and shoot lengths of transformed *N. benthamiana* as compared to non-transformed, **b** Germination rate of transformed *N. benthamiana* as compared to non-transformed, and **c** Growth of transformed and non-transformed *N. benthamiana* in soil pots under greenhouse conditions. Results are the mean values from three independent experiments. Vertical bars indicate SD

Seed germination, and morphological characters were measured to observe the penalty of *ptHR375* and *ptHR941* on transformed *N. benthamiana*. Results have shown that no significant differences were present between non-transformed and transformed *N. benthamiana* **(**Fig. 7**)**. From these results, we can conclude that *ptHR* genes have increased the plant resistance level without affecting its normal germination and morphological characters. No significant penalty was caused by *ptHR* genes in transformed *N. benthamiana*.

### Induced bioactive compounds in *ptHR375* transformed *N. benthamiana*

For the analysis of induced metabolites profile, *ptHR375* transformed *N. benthamiana* was considered and compared with non-transformed *N. benthamiana*. UPLC-QTOF-MS results revealed that a number of specific bioactive compounds were detected in *ptHR375* transformed *N. benthamiana* (Table 1**,** Additional file 1: Figure S5 and Table S5). Among these detected compounds, the contents of Cuelure were present consistently at high amount, and can be considered as the biomarker for *ptHR375* because of its easy detection.

**Table 1:** List of induced bioactive compounds present in *ptHR375* transformed *N. benthamiana*

Compound	Content (μg ribitol equivalent/g of dry weight)	Functions	Reference
Oxytetracycline	235.07 ± 18.19	1) Broad spectrum antibiotic against wide range of bacteria. 2) Used to treat infections caused by Chlamydia and Mycoplasma.	[67]
Cuelure	1776.7 ± 140.53	1) Used for the detection, monitoring and control of fruit fly. 2) Enhance the sex attraction in male fruit flies.	[68, 69]
Allantoin	627.1463 ± 49.27	1) Activates the antioxidant mechanisms in Arabidopsis to increase the tolerance against heavy metals.	[70–72]
Diethylstilbestrol	44.149 ± 3.71	1) Damages the DNA of spermatogonial stem cells and used for the pest management by inducing infertility in male rates.	[73, 74]
1,2-Benzisothiazol-3(2H)-one	795.9675 ± 61.61	1) Derivatives of this compound exhibits antibacterial activities against *Staphylococcus aureus*-ATCC 7000699, and *S. aureus*-ATCC 29213, and antifungal activities against *Candida albicans*, *Cryptococcus neoformans*, *Sporothrix schenckii*, and *C. parapsilosis*.	[75, 76]

## Discussion

In plant resistance breeding, a bottleneck is that the absence and easy to be overcome of plant resistance genes. In this study, we attempted to study the non-self-recognition mechanism between two different plant species, *P. ternata* and *N. benthamiana,* for the development of a strategy to solve the bottleneck of plant resistance breeding. For this purpose, we identified unique DNA sequences from *P. ternata*, which can repeatedly induce non-self-recognition reaction and enhance the basal resistance level of *N. benthamiana*.

*P. ternata* is one of the famous Chinese traditional medicinal herb used to treat a number of diseases. It is widely studied for the extraction of its extracts and identification of its compounds [36]. Until now, there is no study available related to its potential use for the inhibition of phytopathogens. So for this purpose, cDNA library was constructed [37] and screened by using transient expression system. It was found that specific genes from *P. ternata*, can alter the response of *N. benthamiana* plant towards invading pathogens.

Reactive oxygen species are involved in plant defense mechanisms and also play roles in the plant developmental process. Oxidative burst is the early event of plant defense where it generates localized ROS to inhibit the spreading of the pathogen. ROS burst is associated with pathogen/microbes associated molecular patterns (PAMPs/MAMPs) and cause HR [38, 39]. Plant cell wall is the first barrier to the entry of pathogen, and it is modified by the formation of papillae at the site of interaction with invading pathogens. Formation of papillae is the earliest plant defense response at the cellular level, and chemical analysis indicated that callose is the main component of this structure [40–43]. In our study, it was found that *ptHR* genes can activate the ROS burst and callose deposition as early events of a defense response in *N. benthamiana*. This activation of early events of plant defense system indicates that *ptHR375* and *ptHR941* have the potential to inhibit the penetration of potential pathogens and help to maintain the plant vigor against adverse environment.

Antioxidants are the proteinaceous compounds which can alter the internal environment of plant to eliminate the invading pathogen. Some of these antioxidants/enzymes are selected to study their behavior in response to *ptHR375* and *ptHR941* genes. POD prevents the entry of the pathogen into the plant tissue by making it hard, as it is involved in the formation of lignins from monolignols by the process of polymerization [44, 45]. Quinones are the toxic compounds for a variety of microorganisms. PPO plays a significant role in the oxidation of phenolic compounds into Quinones and contributes a part of the resistance against pathogens [6]. SOD is an important enzyme which plays a pivotal role in plant defense mechanisms. It is responsible to protect the plant from ROS burst which causes oxidative damages [46]. In the present study, it has been found that *N. benthamiana* shows an increased level of these antioxidants after the infitration with *ptHR* genes.

Many studies have shown that many invading pathogens can trigger biochemical pathways associated with the expression of pathogenesis-related proteins, such as SA pathway marker genes *PR-1a* and *PR-5* [47, 48]*,* JA/ET pathway marker gene *PDF1.2* [49] and ET pathway marker gene *ERF1* [8]. In plants, induction of *PR-1*, *PR-2*, *PR-5* and *PR-8* is the basic characteristic of SAR, as in TMV-infected tobacco leaf tissue, *PR-1* accounts for the 1% of total leaf protein [50]. Expression of *PDF1.2* is induced by the pathogen attack locally at the site of infection as well as systemically in other non-infected plant parts [51]. *PDF1.2* has also been reported to encode a small protein with antifungal potential [52]. Studies suggested that *ERF1* enhance the resistance against eyespot disease caused by *Rhizoctonia cerealis* in wheat, by the inducing the *PR* genes from ET pathway [53]. It is documented that the cloning of *ERF1-V* from wild species of wheat, *Haynaldia villosa*, can induce the high level resistance against powdery mildew and also improve abiotic stress tolerance [54]. *NPR1*, involved in the cross functioning of SA- and JA-dependent defense pathways, is a key regulator of systemic acquired resistance (SAR) to activate the expression of other pathogenesis-related genes [7, 55]. NPR1 also interacts with the TGA transcription factor members that can bind to the *activator sequence-1* (*as-1*) or the elements which are identified in promoters of *PR-1* genes [56]. PAL is the first enzyme in phenylpropanoid biosynthetic pathway which is involved in plant defense by producing antimicrobial compounds like phytoalexins, lignins and other phenolic compounds to create barriers to pathogens. *PAL* gene is known to be involved in the synthesis of these antimicrobial compounds by providing precursors [7, 57, 58]. It is already reported that ROS burst, especially H_2_O_2_ stimulated by *RBOHB* (*respiratory burst oxidase homolog*) gene, plays an important role in plant defense against biotic and abiotic stresses [59, 60]. It is also reported for the death induction in cells infected with fungus and instantaneously inhibits the free salicylic acid and ethylene to avoid the death in neighboring cells [61]. In this study, positive expression of these genes was observed to induce the resistance in *N. benthamiana*. So it is considered that might be, the mechanism of plant defense response induced by *ptHR375* and *ptHR941* is regulated by SA and JA/ET signal pathways together but still, further investigation is required to make exact signaling pathways clear.

Positively transformed tobacco plants were obtained by using Agrobacterium-mediated leaf disc transformation method. For the selection of positive plants, 3 mg/L bialaphos antibiotic was used as a selection marker, and also gene-specific primers were used for the detection of gene integration after the extraction of genomic DNA. From our results, at T_0_ stage necrotic lesions were observed on initial leaves of transformed plants while at the same time these lesions were not present on non-transformed leaves, later on, these lesions did not appear on T_1_, T_2_ and T_3_ generations. From 1268 genes, we selected *ptHR375* and *ptHR941* two genes, and these two genes can trigger severe responses in *N. benthamiana* with necrotic lesions appeared at the T_0_ stage. However, the transformed tobacco plants can adapt, to some extent, to these two genes and the necrotic lesions disappeared at the T_1_ generation. At the T_1_ generation, although the necrotic lesions disappeared, the resistance level was still significantly increased comparing with non-transformed plants. Why the necrotic lesions disappeared but the foreign transformed *ptHR* genes still play a role in the T_1_ generation? The enhanced *PR* gene expression level (and other evidences like secondary metabolites and resistance against *P. capsici*) at the T_1_ generation may provide a clue but for underlying mechanism it still needs further researches. It was also assumed that at T_0_ stage when *ptHR375* and *ptHR941* genes were not in stable interaction with tobacco genome, development of necrotic lesions was a sign of positive gene integration which was later confirmed by PCR with gene-specific primers.

In plant-microbe interactions, the apoplastic space is the place where many biochemical interactions occur, including the interaction between attacking enzymes and inhibitors of host plant [62, 63]. In plants, hypersensitive response (localized infection) can produce strong defense signals to activate the defense response of the whole plant against upcoming invading pathogens [64]. *NPR1* is a key regulator of systemic acquired resistance (SAR) to protect plants against a variety of pathogens [55]. For the present study, we have used a fungal pathogens, *P. capsici* [33, 63], and found that *ptHR375* and *ptHR941* have potential to inhibit the growth of pathogen and create pathogen independent durable resistance which can be inherited generation after generation. Decreased infestation area of pathogen in transformed *N. benthamiana* lines, which were showing high *PR* genes expression also indicated an obvious correlation between *ptHR* and *PR* genes expression and pathogen resistance.

Heterosis is a phenomena which has been used to explain the improvement of certain characters in F_1_ hybrids of different crops [20]. In our experiments, transformed *N. benthamiana* was crossed with non-transformed *N. benthamiana* to generate F_1_ hybrid. From the analysis of F_1_ hybrid, it was observed that expression of *PR*-genes is significantly up-regulated. There is also an elevated basal resistance level as it was evaluated against *P. capsici*. So from our findings, it can be concluded that heterosis works well to promote F_1_ hybrid disease resistance as it has also been used to boost up the yield potential of different crops [18, 20, 65, 66]. Here when generated F_1_ hybrids, we took *ptHR* gene transformed *N. benthamiana* (T_3_ generation) as the paternal line and non-transformed *N. benthamiana* as the maternal line. The function of *ptHR* gene in the F_1_ hybrids is more or less like that in the *ptHR* gene transformed T_0_ generation. This can explain our result that the fresh F_1_ hybrids have fresh disease resistance. Moreover, the resistance generated by this strategy is not related to any specific pathogen, and it has a broad spectrom resistance.This finding may change the mode of plant resistance breeding because the F_1_ hybrids produced by using this strategy can be used to develop different and durable resistant cultivars to manage field crop diseases.

Secondary metabolites profile of *ptHR375* transformed *N. benthamiana* revealed the presence of some bioactive compounds which were not detected in non-transformed *N. benthamiana*. These bioactive compounds are reported for their biological activities as Oxytetracycline reported to treat Chlamydia and Mycoplasma infection [67]. Cuelure was reported for the monitoring of fruit fly [68, 69] and Allantoin have the potential to activate the plant system against heavy metals [70–72]. For the pest management, Diethylstilbestrol was used because it can make male rates infertile [73, 74], and the derivatives of 1,2-Benzisothiazol-3(2H)-one are strong antibacterial and antifungal agents [75, 76]. It can be considered that induction of these bioactive compounds also confers role to contribute resistance in *ptHR375* transformed *N. benthamiana*.

From this research, we can conclude that non-self-recognition between two different plant species is an important mechanism which works to initiate the immune responses against pathogens. As these *ptHR* genes from *P. ternata* also have the ability to initiate HR symptoms on other important crops like *L. esculentum* and *G. hirsutum* (Additional file 1: Figure S2), so in future, these genes can be used to create resistance in other economically important crops. Here we tried to prove that when heterologous genes from *P. ternata* were introduced into *N. bentamiana* plant, its basal resistance level was increased up to several folds. This concept has opened a new window of plant-microbe interaction research which can be helpful to develop a new genetic system to introduce new potential resistant cultivars for the betterment of sustainable agriculture. In the course of crop domestication and improvement, genetic diversity was reduced, and plant resistance was lost. Here introducing one kind of *ptHR* genes into modern crop plant, the plant may get into a prime state, and the loss of natural resistances may get compensation. In the next researches, based on these *ptHR* genes, we will evaluate, to what extent, the lost natural resistances from wild to cultivated crops can get compensated.

## Conclusion

Two *ptHR* genes, *ptHR375* and *ptHR941* were identified from *P. ternata* with the potential to trigger HR. Both genes are involved in different resistance pathways. *ptHR375* involved in the activation of JA/ET pathway, while *ptHR941* also in the SA pathway. These heterologous plant genes can activate disease resistance in *N. benthamiana* and furthermore, by generating F_1_ hybrids, fresh pathogen independent plant resistance can be obtained. Feasibility of this hybridization design may help to improve the breeding strategies for the development of durable resistance in economically important crops.

## Additional file

Additional file 1: **Table S1.** Primers used for qRT-PCR**. Figure S1.** Agarose gel of cDNA inserts. M, 100 bp marker. **Figure S2.** Hypersensitive response induced by *ptHR* genes on *L. esculentum, G. hirustum, N. benthamiana* and *P. ternata* leaves. **Table S2.** NCBI blast results showing homology with known sequences. **Table S3.** NCBI blast results showing homology with transcription factors. **Table S4.** NCBI blast results showing no homology with known sequences. **Figure S3.** Relative expression levels of pathogenesis-related genes in *N. benthamiana.* **Figure S4.** Relative expression levels of *ptHR941* and *ptHR375* genes in transformed *N. benthamiana*. **Figure S5.** Mass spectra for the induced bioactive compounds detected in *ptHR375* transformed *N. benthamiana*. **Table S5.** List of induced bioactive compounds present in *ptHR375* transformed *N. benthamiana. (PDF 1033 kb)*

## Abbreviations

hpi: Hours post infiltration
HR: Hypersensitive response
POD: Peroxidase
PPO: Poly phenol oxidase
*PR*-genes: Pathogenesis related genes
*ptHR*: *Pinellia ternata hypersensitive response*
qRT-PCR: Quantitative reverse transcription PCR
ROS: Reactive oxygen species
SOD: Superoxide dismutase
