# Supplementary material for: Enhanced Nicotiana benthamiana immune responses caused by heterologous plant genes from Pinellia ternata
Source: BMC Plant Biol. 2018 Dec 17;18:357. doi: 10.1186/s12870-018-1598-5 (PMC6296014; doi:10.1186/s12870-018-1598-5)
Supplement: Supplementary file 1 — Table S1. Primers used for qRT-PCR. Figure S1. Agarose gel of cDNA inserts. M, 100 bp marker. Figure S2. Hypersensitive response induced by ptHR genes on L. esculentum, G. hirustum, N. benthamiana and P. ternata leaves. Table S2. NCBI blast results showing homology with known sequences. Table S3. NCBI blast results showing homology with transcription factors. Table S4. NCBI blast results showing no homology with known sequences. Figure S3. Relative expression levels of pathogenesis-related genes in N. benthamiana. Figure S4. Relative expression levels of ptHR941 and ptHR375 genes in transformed N. benthamiana. Figure S5. Mass spectra for the induced bioactive compounds detected in ptHR375 transformed N. benthamiana. Table S5. List of induced bioactive compounds present in ptHR375 transformed N. benthamiana. (PDF 1033 kb) [file 12870_2018_1598_MOESM1_ESM.pdf]

Supplementary material

Enhanced *Nicotiana benthamiana* immune responses caused by heterologous plant genes from *Pinellia ternata*

Hafiz Muhammad Khalid Abbas, Jingshu Xiang, Zahoor Ahmad, Lilin Wang, Wubei Dong*

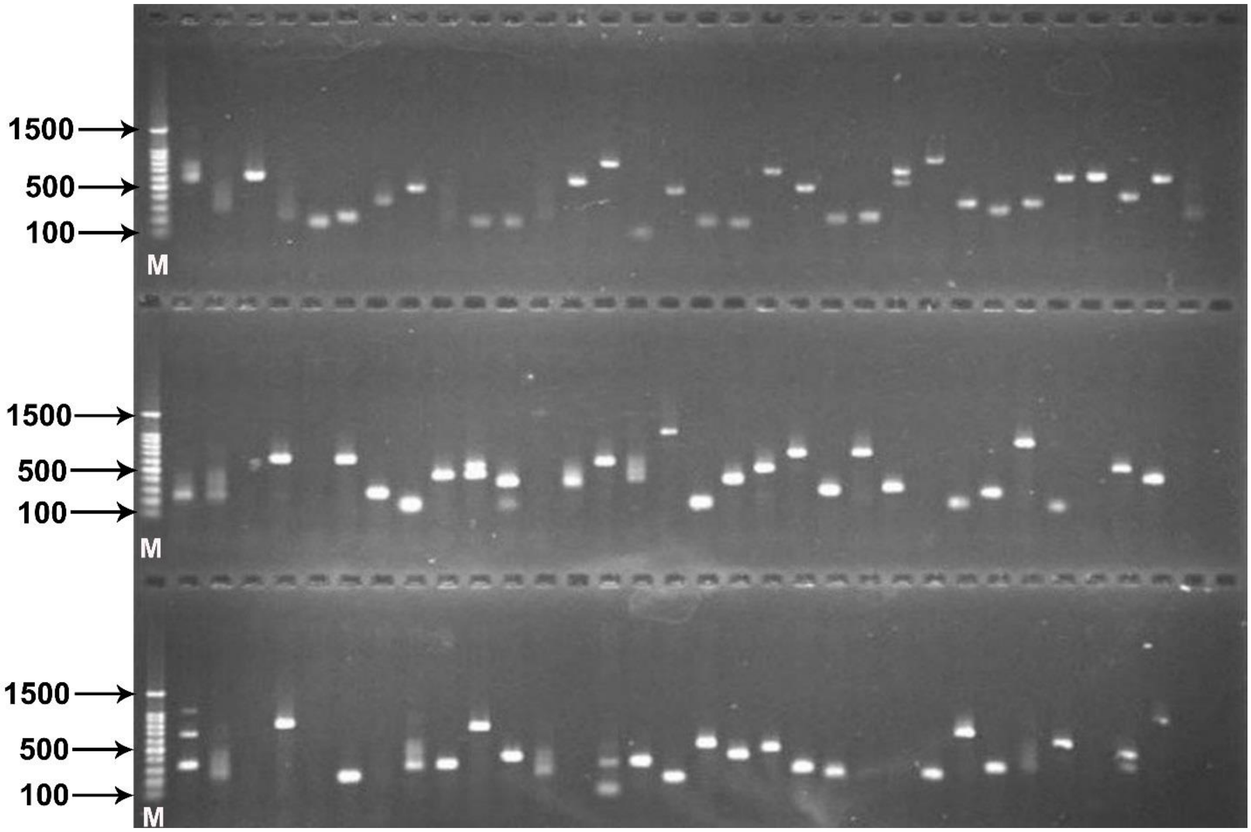

Fig. S1. Agarose gel of cDNA inserts. **M**, 100bp marker.

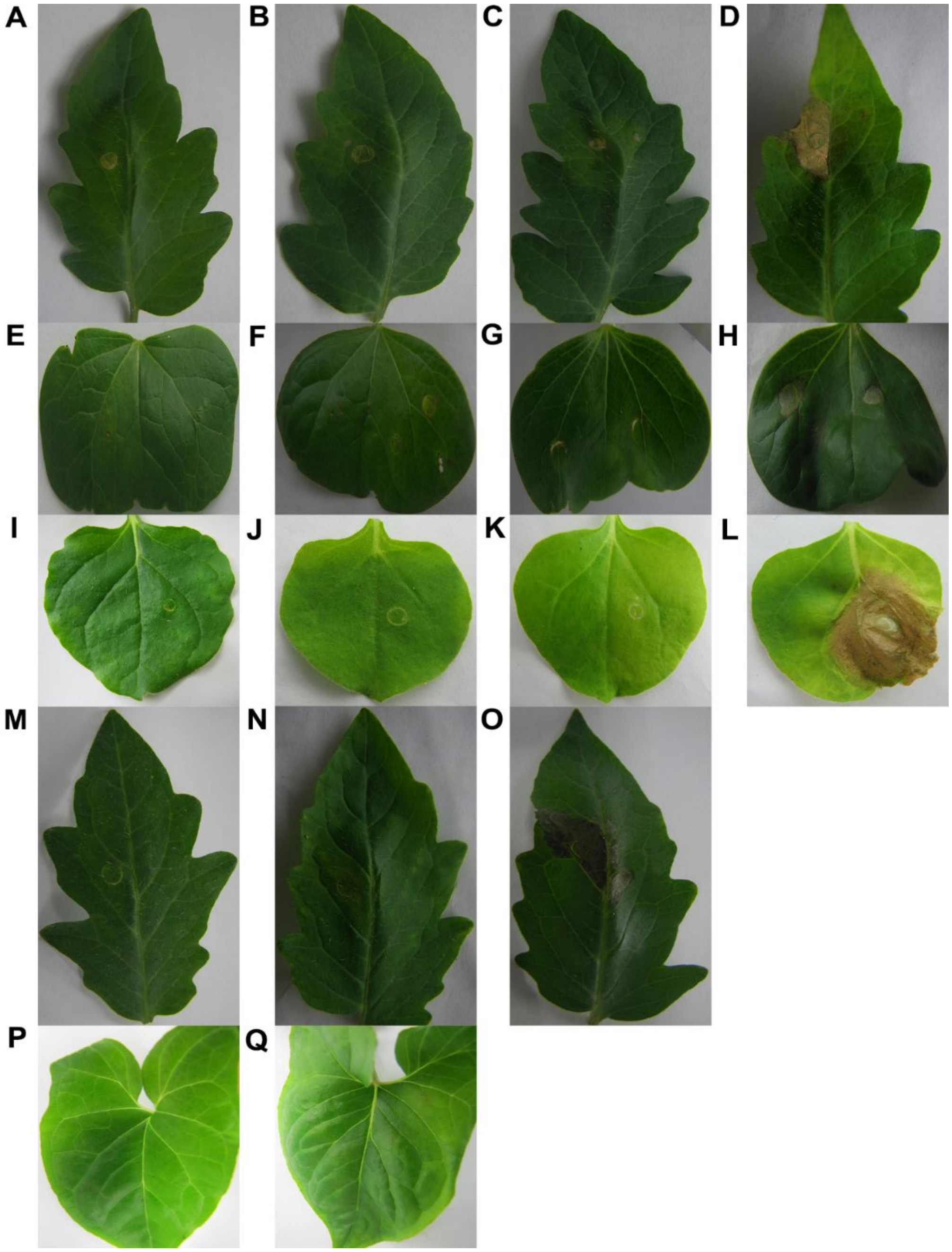

Fig. S2. Hypersensitive response induced by *ptHR* genes on *L. esculentum, G. hirustum, N. benthamiana* and *P. ternata* leaves.

L. esculentum and *G. hirustum* and *N. benthamiana* leaves were infiltrated with EHA105 Agrobacterium strain, empty vector and *ptHR* genes. Pictures were taken at 48 h of post infiltration. **a-d** *L. esculentum* leaves treated with **a** Agrobacterium EHA105 strain, **b** pTRV empty vector, **c** $pTRV_1$ as control and **d** *ptHR941*. **e-h** *G. hirustum* leaves treated with **e** Agrobacterium EHA105 strain, **f** pTRV empty vector, **g** $pTRV_1$ as control and **h** *ptHR941*. **i-l** *N. benthamiana* leaves treated

with **i** buffer, **j** Agrobacterium EHA105 strain, **k** pCAMBIA3301 and **l** *ptHR941*. **m-o** *L. esculentum* leaves treated with **m** Agrobacterium EHA105 strain, **n** pCAMBIA3301 empty vector and **o** *ptHR941*. *P. ternata* leaves were infiltrated with empty vector and *ptHR* genes. **p-q** *P. ternata* leaves treated with **p** pCAMBIA3301 empty vector and **q** *ptHR941*. Each experiment was repeated three times and each time same results were observed. Representative pictures are shown here.

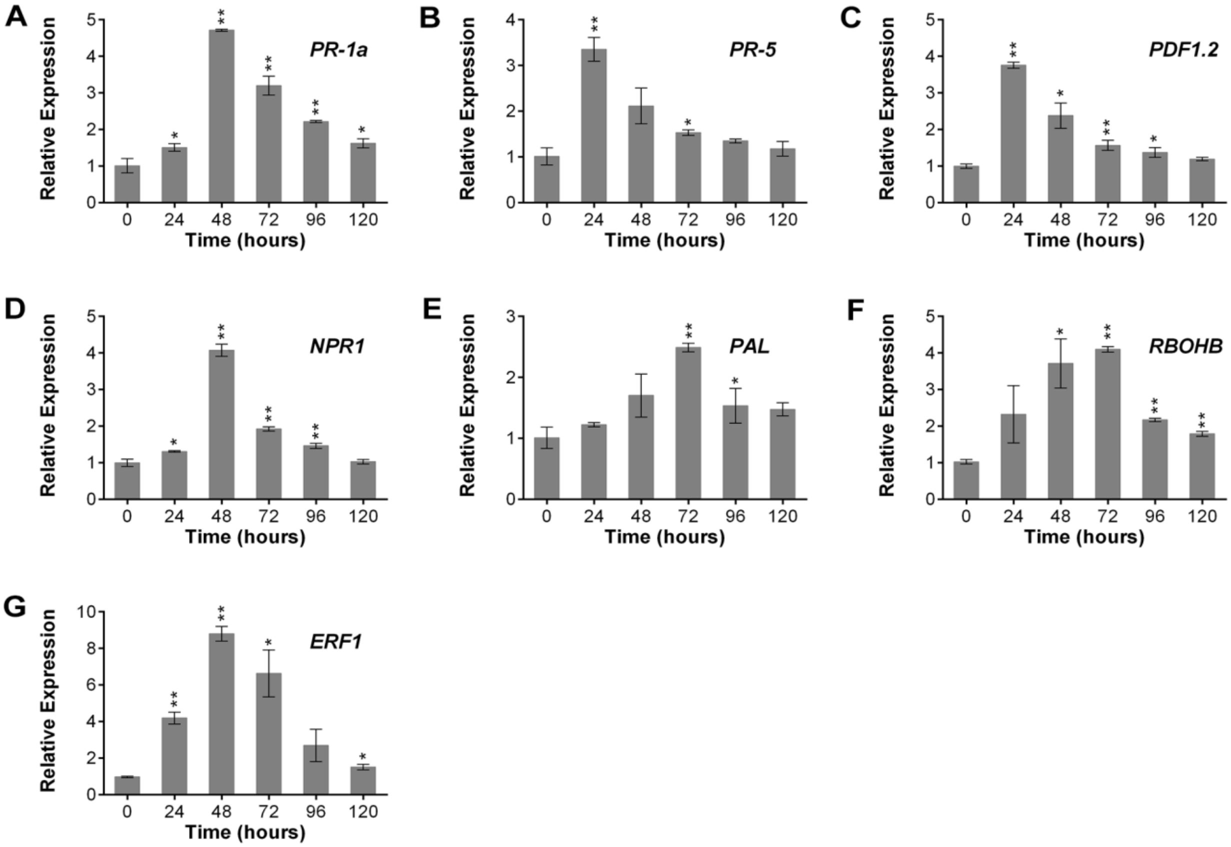

Fig. S3. Relative expression levels of pathogenesis-related genes in *N. benthamiana*.

N. benthamiana leaves were infiltrated with *ptHR375* and an empty vector as control for RT-qPCR analysis. Leaves infiltrated with empty vector were used as control for relative quantification of gene expression. *EF-1α* was used as indigenous control. **a-g** Relative expression levels of, **a** *PR-1a* vs control, **b** *PR-5* vs control, **c** *PDF1.2* vs control, **d** *NPR1* vs control, **e** *PAL* vs control, **f** *RBOHB* vs control and **g** *ERF1* vs control. Significance was determined by t-test: *P<0.05, **P<0.01. Results are the mean values from three independent experiments. Vertical bars indicate SD.

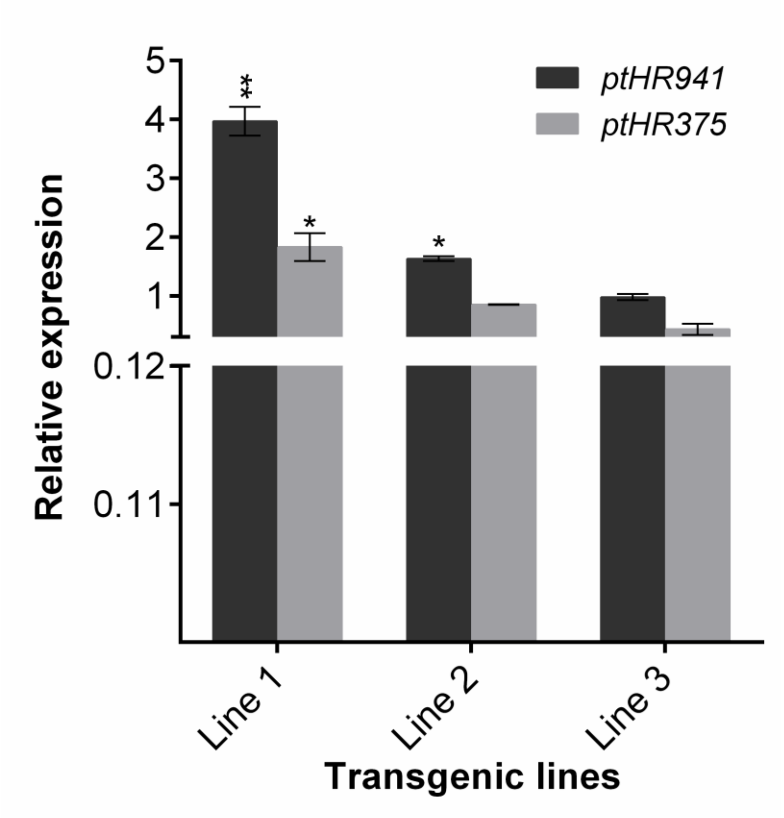

Fig. S4. Relative expression levels of *ptHR941* and *ptHR375* genes in transformed *N. benthamiana*.

Leaves were sampled from transformed *N. benthamiana* (T_3) to extract total RNA for RT-qPCR analysis. Transformed (T_2) *N. benthamiana* was used as control for relative quantification of gene expression. *EF-1α* was used as internal control. Figure showing the relative expression of *ptHR941* and *ptHR375* genes in transformed *N. benthamiana* (T_3) compared with control. Results are the mean values from three independent experiments. Vertical bars indicate SD. Significance was determined by t-test: *P<0.05, **P<0.01.

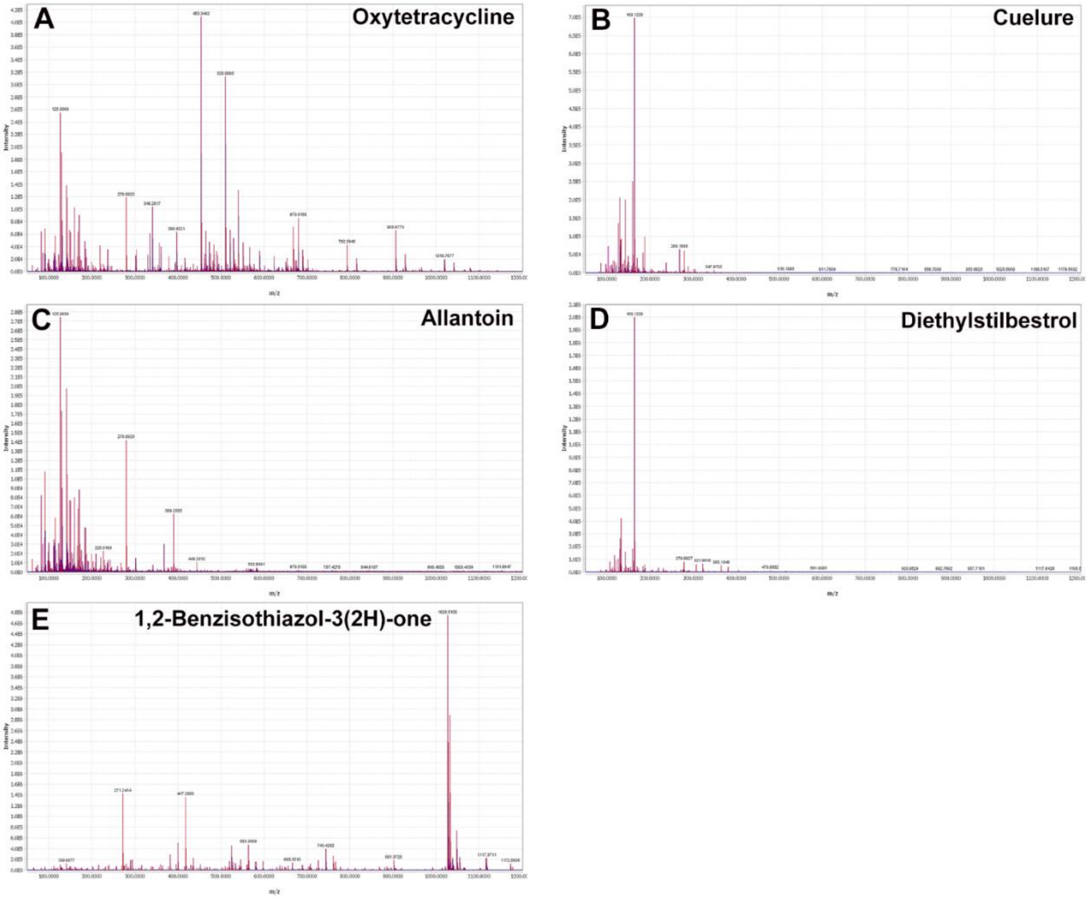

Fig. S5. Mass spectra for the induced bioactive compounds detected in *ptHR375* transformed *N. benthamiana*. **a** Oxytetracycline, **b** Cuelure, **c** Allantoin, **d** Diethylstilbestrol and **e** 1,2-Benzisothiazol-3(2H)-one.

Table S1 Primers used for qRT-PCR**.**

**Gene**	**Forward Primer（5’—3’）**	**Reverse Primer（5’—3’）**
EF-1α	TGTGATGTTTTTGTTCGGTCTTTAA	TCAAAAGAAAATGCAGACAGACTCA
PR-1a	CCTCGTACATTCTCATGGTCAAT	CCATTGTTACACTGAACCCTAGC
PDF1.2	GGAAATGGCAAACTCCATGCG	ATCCTTCGGTCAGACAAACG
NPR1	ACATCAGCGGAAGCAGTAG	GTCGGCGAAGTAGTCAAAC
PAL	GTTATGCTCTTAGAACGTCGCCC	CCGTGTAATGCCTTGTTTCTTGA
RBOHB	TTTTCTCTGAGGTTTGCCAGCCACCA	GCCTTCATGTTGTTGACAATGTCTTT
ERF1	GGCGAATTTTCCGGGAGACT	GGCTCCGATTTTACTTCGCC
ptHR941	TCAGGTTATGCCGTGCTCG	AAGGAAGCGTCTACAGGGAG
ptHR375	CAGGATCTCCACGCCGAAGC	AGCAGCTTCTACTTGAATCGAT

Table S2 NCBI blast results showing homology with known sequences.

Gene	Length (bp)	Homology	Identity (%)
ptHR274	137	Echinostoma caproni genome assembly E_caproni_Egypt, scaffold ECPE_scaffold0025465	89
ptHR284	410	Spirodela polyrhiza strain 9509 chromosome 1 sequence	77
ptHR298	395	Spirodela polyrhiza strain 9509 chromosome 2 sequence	79
ptHR310	80	Zantedeschia aethiopica rubisco activase (rca4) mRNA, partial cds	94
ptHR317	408	Spirodela polyrhiza strain 9509 chromosome 14 sequence	88
ptHR359	346	PREDICTED: Asparagus officinalis ATP-dependent Clp protease proteolytic subunit-related protein 1, chloroplastic (LOC109835634), mRNA	78
ptHR375	254	PREDICTED: Daucus carota subsp. sativus metallothionein-like protein type 3 (LOC108220108), mRNA	88
ptHR388	763	PREDICTED: Phoenix dactylifera UV-B-induced protein At3g17800, chloroplastic-like (LOC103713309), mRNA	76
ptHR449	173	Spirodela polyrhiza strain 9509 chromosome 15 sequence	86
ptHR478	498	Oryza sativa Indica Group cultivar Shuhui498 chromosome 3 sequence	79
ptHR615	711	Spirodela polyrhiza strain 9509 chromosome 7 sequence	88
ptHR620	476	PREDICTED: Musa acuminata subsp. malaccensis 60S ribosomal protein L21-1 (LOC103980608), mRNA	86
ptHR805	547	PREDICTED: Musa acuminata subsp. malaccensis uncharacterized LOC103976420 (LOC103976420), mRNA	88
ptHR812	414	PREDICTED: Phoenix dactylifera glutamate dehydrogenase 2-like (LOC103710782), mRNA	82
ptHR813	94	PREDICTED: Eucalyptus grandis 5-methyltetrahydropteroyltriglutamate--homocysteine methyltransferase (LOC104443139), mRNA	96

ptHR830	346	PREDICTED: Asparagus officinalis ATP-dependent Clp protease proteolytic subunit-related protein 1, chloroplastic (LOC109835634), mRNA	78
ptHR844	465	Phaseolus vulgaris clone BE5D669 mitochondrial import inner membrane translocase subunit tim17 mRNA, complete cds; nuclear gene for mitochondrial product	88
ptHR849	232	Mouse DNA sequence from clone RP23-169H17 on chromosome 2, complete sequence	90
ptHR897	602	PREDICTED: Elaeis guineensis RGG repeats nuclear RNA binding protein A (LOC105048011), mRNA	70
ptHR941	190	PREDICTED: Gossypium raimondii sulfite reductase [ferredoxin], chloroplastic-like (LOC105794174), mRNA	76
ptHR1028	278	PREDICTED: Elaeis guineensis 125 kDa kinesin-related protein-like (LOC105044532), mRNA	83
ptHR1041	533	Phyllostachys edulis cDNA clone: bbasst002k07, full insert sequence	86
ptHR1067	126	Spirodela polyrhiza strain 9509 chromosome 12 sequence	82
ptHR1070	971	Spirodela polyrhiza strain 9509 chromosome 2 sequence	75
ptHR1092	186	PREDICTED: Zea mays uncharacterized LOC103636543 (LOC103636543), ncRNA	77

Table S3 NCBI blast results showing homology with transcription factors.

**Gene**	**Length (bp)**	**Homology**	**Identity (%)**
ptHR69	177	PREDICTED: Ananas comosus nuclear transcription factor Y subunit B-3-like (LOC109711511), mRNA	100
ptHR293	749	PREDICTED: *Setaria italica* probable WRKY transcription factor 33 (LOC101782717), mRNA	76
ptHR759	363	PREDICTED: *Musa acuminata* subsp. malaccensis NAC domain-containing protein 21/22-like (LOC103995621), mRNA	89
ptHR1015	123	PREDICTED: *Nelumbo nucifera* probable sucrose-phosphate synthase 3 (LOC104602206), transcript variant X2, mRNA	92

ptHR1028	278	PREDICTED: *Asparagus officinalis* kinesin-like protein KIN-5A (LOC109848469), transcript variant X1, mRNA	84

Table S4 NCBI blast results showing no homology with known sequences.

Gene	Length (bp)	Gene	Length (bp)
ptHR40	47	***ptHR772***	607
ptHR47	166	***ptHR788***	609
ptHR99	207	***ptHR829***	129
ptHR2-20	310	***ptHR831***	60
ptHR268	203	***ptHR835***	47
ptHR292	196	***ptHR841***	530
ptHR519	166	***ptHR917***	91
ptHR601	89	***ptHR943***	47
ptHR602	47	***ptHR1124***	91
ptHR612	60		

Table S5 List of induced bioactive compounds present in *ptHR375* transformed *N. benthamiana*

Compound	Retention time (min)	m/z $[M+H]^+$	Molecular weight (g/mol)
Oxytetracycline	5.31	460.83	460.43
Cuelure	1.20	103.95	206.24
Allantoin	6.04	86.98	158.11
Diethylstilbestrol	0.91	466.87	268.36
1,2-Benzisothiazol-3(2H)-one	6.61	188.95	151.18
